# Effects of proinflammatory cytokines and programmed cell death on cognitive domains in older age patients with bipolar disorder

**DOI:** 10.1186/s12991-025-00591-9

**Published:** 2025-08-12

**Authors:** Pei-Ying Lee, Chih Chiang Chiu, Po-Hsiu Kuo, Cho-Yin Huang, Shang-Ying Tsai, Chian-Jue Kuo, Wen-Yin Chen

**Affiliations:** 1 Department of Psychiatry, Taipei City Psychiatric Center, Taipei City Hospital, Taipei, Taiwan; 2 School of Medicine, College of Medicine, Fu Jen Catholic University, New Taipei, Taiwan; 3 Department of Psychiatry, School of Medicine, College of Medicine, Taipei Medical University, Taipei, Taiwan; 4 Institute of Health Policy and Management, College of Public Health,, National Taiwan University, Taipei, Taiwan; 5 Department of Public Health, College of Public Health, National Taiwan University, Taipei, Taiwan; 7 Psychiatric Research Center, Taipei Medical University Hospital, Taipei, Taiwan

**Keywords:** Older age bipolar disorders (OABD), Neuroaxonal integrity, Programmed cell death, Neuroinflammatory, Cytokines

## Abstract

**Objectives:**

Proinflammatory cytokines are linked to cognitive deficits in bipolar disorder (BD). The programmed cell death (PD) pathway, involved in immune regulation, may impact mood disorders and dementia. Older age BD (OABD) patients face a heightened risk of cognitive decline, yet studies exploring the underlying mechanisms in this population are scarce. Aim of this study is to investigate proinflammatory cytokines and the PD pathway in OABD, for their correlation with clinical features and neuroaxonal integrity, and the impact on cognitive domains.

**Methods:**

Eighty-seven euthymic OABD patients were assessed using the Brief Assessment of Cognition in Affective Disorders. We measured CRP, IL-6, TNF-α, TNF-R1, TNF-R2, PD-1, and PD-L1. Neurofilament light chain (NfL) was used to gauge neuroaxonal integrity. Associations between cytokines, PD-1/PD-L1, and cognition were examined using linear regression models.

**Results:**

The average age of the OABD patients was 59.64 with a mean illness duration of 27.19 years. NfL levels positively correlated with TNF-R2 levels. Regression analysis revealed a negative association between TNF-R1 and motor speed and verbal fluency, while TNF-R2 showed positive associations with these cognitive domains. PD-1 was negatively associated with composite score, especially in motor speed and working memory, while PD-L1 was positively associated with executive function.

**Conclusion:**

This is the first study to simultaneously examine the proinflammatory system and the PD-1/PD-L1 pathway in a clinical OABD sample, with findings suggesting that both systems impact cognitive function in OABD patients. Further research is needed to explore the neuroinflammatory mechanisms underlying BD’s neurodegenerative course.

**Supplementary Information:**

The online version contains supplementary material available at 10.1186/s12991-025-00591-9.

## Introduction

Bipolar disorder (BD) is a chronic psychiatric disorder characterized by manic and depressive mood episodes accompanied by recurrent relapses and significant functional impairment. A core feature of BD is cognitive impairment, which is present across all mood states [1–4]. Among the various cognitive domains, verbal memory and executive function are the most consistently affected, and these impairments may partially explain the functional limitations associated with BD [5]. Moreover, older adults with BD face a heightened risk of cognitive decline that differs from typical age-related changes [6–9].

Cognitive impairment in BD may result from distinct mechanisms across the lifespan. In younger individuals, the neurodevelopmental hypothesis suggests that early genetic, environmental, or neurobiological disruptions interfere with brain maturation, leading to lasting vulnerabilities in executive function, attention, and working memory [10, 11]. These impairments may stem from altered synaptic pruning, disrupted cortical development, and impaired myelination [11]. In contrast, during the later stages of life in older adults with bipolar disorder (OABD), cognitive decline is more commonly exacerbated by neurodegenerative processes. Although several potential mechanisms have been proposed—including dysregulation of the dopaminergic and glutamatergic systems, mitochondrial dysfunction, and oxidative stress [12, 13], current evidence largely supports inflammation as a primary underlying process [2, 14, 15]. Nevertheless, the precise mechanisms underlying cognitive deficits—particularly in OABD—have yet to be definitively determined.

In patients with BD, elevated levels of cytokines—including tumor necrosis factor receptor 1 (TNFR1), tumor necrosis factor-alpha (TNF-α), interferon (IFN), and interleukins 2, 4, 6, 10, and 17 (IL-2, IL-4, IL-6, IL-10, IL-17)—have been documented [16–21]. Among these, the pro-inflammatory cytokines TNF-α, TNFR1, and IL-6 have been consistently reported to be associated with BD in independent studies [21–23]. Additionally, progressive neuropathological changes, various mood phases, and the overall disease course in BD may be associated with alterations in inflammatory cytokines [24, 25]. For instance, studies on BD have shown a positive association between elevated TNF-α levels and both illness progression and mood episode fluctuations [26, 27]. These findings suggest that increased inflammatory activity is related to progression of the disorder. Among the dysregulated markers, recent evidence further suggests an association between cognitive impairment and abnormal levels of C-reactive protein (CRP), interleukin-1 receptor antagonist (IL-1RA), IL-6, and TNF-α, along with their respective receptors [15]. Moreover, elevated peripheral inflammatory markers are most frequently associated with decreased structural or functional measures on neuroimaging [28]. However, most studies investigating the association between inflammation and cognitive function in BD have been conducted in younger adult populations; there was only one study that observed a negative correlation between IL-1 receptor antagonists and global cognitive function in OABD [29].

TNF-α is a cytokine with diverse biological roles, mediated via TNFR1 and TNF receptor 2 (TNFR2) signaling pathways [30]. TNF-α can orchestrate various cellular responses, including activation of inflammatory gene expression, promotion of cellular proliferation and differentiation, and initiation of programmed cell death (PD) pathways, such as apoptosis and necroptosis [31]. In an acute episode of BD, pro-inflammatory cytokines activate a compensatory immune-regulatory reflex system, and even after the acute episode subsides, the imbalance within the immune system persists [32]. Programmed cell death protein 1 (PD-1), a membrane protein expressed on T cells, contributes to apoptosis and serves as a binding partner to programmed death-ligand 1 (PD-L1), transmitting inhibitory signals that regulate the balance among T-cell activation, immune tolerance, and prevention of immunopathology [33–35]. Dysregulation of the PD pathway has been associated with various brain disorders, and emerging evidence suggests its involvement in neuroinflammation related to neurodegenerative and affective disorders [36–38]; however, this pathway has not been directly studied in relation to OABD.

Emerging evidence suggests that neuroinflammatory processes contribute to cognitive impairments across various neuropsychiatric disorders, potentially with domain-specific effects. For exapmle, in Alzheimer’s disease, microglial activation has been strongly linked to memory dysfunction, while in multiple sclerosis, hippocampal synaptic phagocytosis has been associated with deficits in learning and working memory [39–41]. Similarly, elevated levels of pro-inflammatory cytokines such as IL-6 and TNF-α have been observed in patients with severe mental illness, correlating with impairments in attention and executive functioning [42, 43]. However, little is currently known about the inflammatory profiles in OABD and their potential associations with specific cognitive domains. We hypothesize that elevated levels of pro-inflammatory markers and dysregulation of the PD pathway may further disrupt neural circuits involved in cognitive regulation in BD, thereby contributing to the diverse cognitive deficits observed. Investigating these mechanisms in OABD could provide valuable insights into the disorder’s pathophysiology and inform the development of targeted interventions.

This study aims to concurrently examine pro-inflammatory cytokine levels and the programmed cell death (PD) pathway in individuals with OABD, to explore their correlations with clinical characteristics and neuroaxonal integrity, and to assess their associations across multiple cognitive domains.

## Methods

### Participants

In this cross-sectional study, we recruited individuals diagnosed with bipolar I disorder from the outpatient clinic of a tertiary psychiatric hospital, based on the Diagnostic and Statistical Manual of Mental Disorders, Fifth Edition (DSM-5). Although older adults are often defined as those aged ≥ 60 or 65, the ISBD Task Force recommends using age 50 as the cutoff in BD research, due to higher medical comorbidities and reduced life expectancy in this population [44]. This definition helps minimize survivorship bias and better reflect the clinical features of aging in BD. Accordingly, our study included individuals aged 50 and above. We excluded individuals with specific issues: [1] a known substance use disorder (with the exception of a nicotine use disorder); [2] any disorder with neurological symptoms or complications, such as brain injury or stroke; [3] an ongoing physical condition/illness, such as renal impairment, hepatic failure, or pregnancy; [4] a previous diagnosis of intellectual disability, schizophrenia, and/or schizoaffective disorders; and/or [5] inability to complete the standard clinical assessment or provide informed consent. Information regarding psychiatric comorbidities and the exclusion criteria was obtained from patients’ medical records and psychiatrist interviews. Individuals were also required to be euthymic and on stable medication (no change in psychotropic medications in the previous one month). The authors assert all procedures involving human subjects were approved by the Research Ethics Committee of Taipei City Hospital (TCHIRB-11201001). Written informed consent was obtained from all patients.

### Measurements

#### Demographic and clinical data, cardiovascular risk and mood symptoms

Demographic data, including age, sex, marital status, family history of psychiatric disorders, smoking habits, alcohol consumption, physical comorbidities, body weight/height, and year of education were collected at the time of enrollment. Participant clinical characteristics included number of affective episodes (total, manic, and major depressive), number of episodes with psychotic features, number of episodes requiring admission, and age of illness onset. The psychopharmacological medications the patients used at the time of assessment were recorded and converted to a defined daily dose (DDD). A DDD, as defined by the World Health Organization [45], is a unit of measurement that represents the assumed average maintenance dose per day of a drug when used for its main indication in adults. This standardized metric is commonly used to facilitate comparisons of drug consumption. The Framingham risk score (FRS score) predicts the risk of cardiovascular disease, and the 10-year risk was measured using the 2018 Prevention Guidelines Cardiovascular Risk Calculator [46] which included variables concerning sex, age, total cholesterol levels, high-density lipoprotein (HDL) levels, systolic blood pressure, smoking habits, diabetes, and known vascular diseases. Mood symptoms were obtained through clinician-administered measures with the 17-item Hamilton Depression Rating Scale (HAM-D) and the Young Mania Rating Scale (YMRS). We defined euthymia as both HAM-D and YMRS ≤ 8.

### Cognitive measurements

The premorbid intelligence quotient (IQ) was estimated by a licensed psychologist who used the Adult Reading Test for the Wechsler Adult Intelligence Scale. The enrolled patients were assessed using the Brief Assessment of Cognition in Affective Disorders (BAC-A), which has extensively been used as a quick and reliable cognitive assessment of patients with a wide range of clinical affective disorders [47]. The BAC-A usually be administered within 35–60 min. The assessment measures affective memory and emotional inhibition through the Affective Auditory Verbal Learning Test and Emotional Stroop Task. We used BAC-A to measure six standard neurocognitive domains: [1] working memory (Digit Sequencing Task) [2], motor speed (Token Motor Task) [3], verbal fluency (Category Instances and Controlled Oral Word Association Test) [4], processing speed (Symbol Coding) [5], verbal memory (List Learning), and [6] executive function (Tower of London), through a comparison with norm references and then convert to standardized z-score [48]. The criterion and construct validity for each test of cognitive impairment as well as the sensitivity of these tests to changes in cognition have been demonstrated in the literature, in addition, each test has also been demonstrated to be valid for use in different cultures and language groups [48].

### Biomarker measurements

Informed by the literature review presented in the introduction, this study analyzed the following pro-inflammatory cytokines: IL-6, TNF-α, TNF-R1, TNF-R2, and CRP. To represent the PD pathway, PD-1 and PD-L1 levels were measured. Neurofilament light chain (NfL) is a well-established, sensitive biomarker of neuroaxonal damage and is widely used to assess neural injury across various neurological disorders [49–52]. Accordingly, peripheral NfL levels were used in this study as an indicator of neuroaxonal integrity.

The patients were subject fasting blood sampling in the early morning on the same day of cognitive assessment. A venous blood sample of 10 mL was taken from each participant, and the plasma was separated and stored at − 80 °C until analyses were conducted. According to the manufacturer’s instructions, plasma levels of CRP, IL-6, TNF-R1, TNF-R2, TNF-α, PD-1 and PD-L1 were measured in duplicate using enzyme-linked immunosorbent assays (ELISAs) with commercially available kits. Peripheral NfL levels were measured using Quanterix SiMoA^®^ assay following the manufacturer’s standard procedures, which consisted of a digital immunoassay with the lower limit of detection as 0.104 pg/mL.

### Statistical analysis

A descriptive analysis was used to present the demographic and clinical characteristics of the enrolled OABD patients by sex. Chi-squared and Student’s *t*-tests were used for assessing categorical and continuous variables, respectively. The normality of data distributions was evaluated using the Kolmogorov–Smirnov test. For data not normally distributed, the nonparametric Wilcoxon rank-sum test was applied. Pearson’s correlation analyses were conducted to examine associations between clinical characteristics and levels of pro-inflammatory cytokines, C-reactive protein (CRP), and PD-1/PD-L1, as well as between clinical characteristics and cognitive outcomes. To reduce the risk of false positives from multiple comparisons, false discovery rate (FDR) correction was applied. Finally, linear regression models were employed to identify the independent effects of these biomarkers on specific cognitive domains. In regression analyses, we report unstandardized beta coefficients (B), as they offer direct interpretability by reflecting the actual effect size in real-world units, making them more useful for clinical relevance. All analyses were conducted using IBM SPSS Statistics (Version 24), and the significance level was set at p-value < 0.05.

## Results

### Patient characteristics and cognitive function

A total of 57 female and 30 male patients were recruited for the study. The mean age of patients with OABD was 59.64 years, with an average illness duration of 27.19 years and a mean total number of episodes of 9.54. Demographic and clinical characteristics are summarized in Table 1. All participants had been on stable psychopharmacological treatment for at least one month prior to the study. The mean score on the HAM-D was 2.46, while the mean score on the YMRS was 2.66. Mean HAM-D scores were 1.80 in males and 2.77 in females, with females exhibiting significantly higher scores (*p* = 0.018). Among the participants, 10.34% had comorbid diabetes mellitus, 13.79% had hypertension or coronary artery disease, 9.20% had hepatitis B or C infections, and 4.60% had thyroid disease. Regarding psychoactive medications, the mean DDD was 0.75 for second-generation antipsychotics (SGAs), 0.50 for mood stabilizers, 0.58 for benzodiazepines (BZDs), and 0.01 for antidepressants. Smoking and alcohol consumption were significantly more prevalent among male patients. No statistically significant differences were observed in the levels of pro-inflammatory cytokines or PD system biomarkers between male and female participants. In the BAC-A assessment, the mean Z score of composite cognition was − 2.03 for males and − 2.64 for females. No significant sex differences were detected in individual cognitive domains (Supplemental Table 1).

**Table 1:** Socio-demographic and clinical characteristics for the OABD

Characteristics, mean (SD)	Male (*N* = 30)	Female (*N* = 57)	*P*-value	Total group (*N* = 87)
Age	58.60(5.89)	60.12 (6.56)	0.290	59.64 (6.37)
Married, n (%)	15 (50.00)	22 (38.59)	0.300	37 (42.53)
With smoking habit, n (%)	10 (33.33)	6 (10.53)	0.012*	16 (18.39)
With alcohol use habit, n (%)	8 (26.67)	3 (5.26)	0.009**	11 (12.64)
Physical comorbidity, n (%) DM, n HTN/CAD, n HBV/HCV, n Thyroid disease, n	10 (33.33) 5 4 4 0	13 (22.81) 6 8 4 4	0.601	23 (26.44) 11 12 8 4
Onset of age	30.96(12.72)	33.36 (13.23)	0.450	32.61 (11.06)
Duration of illness	27.19 (11.38)	27.20(11.01)	0.390	27.19 (11.08)
Education years	12.44 (3.94)	11.12(4.38)	0.204	11.82 (4.14)
Estimated premorbid IQ	101.50 (7.86)	88.91(18.64)	0.060	96.90 (15.59)
Number of total episodes	7.78(4.50)	9.23 (4.72)	0.205	9.54 (4.66)
Number of manic episodes	5.89(4.26)	6.47 (3.99)	0.569	6.09 (4.02)
Number depressive episodes	1.85(1.46)	2.60 (2.68)	0.134	4.34 (3.34)
Number psychotic episodes	3.56(2.72)	3.18 (3.23)	0.618	3.32 (3.06)
Number of episodes requiring admission	5.41(5.21)	7.09 (6.34)	0.252	6.41 (5.96)
BMI	22.95(5.91)	24.12(5.58)	0.436	23.67 (5.69)
HAM-D	1.80 (1.81)	2.77 (1.69)	0.018*	2.46(1.80)
YMRS	2.27 (2.05)	2.81 (2.37)	0.294	2.66(2.28)
*Pro-inflammatory biomarkers*				
CRP, mg/ml	2.66(2.98)	2.68(3.71)	0.983	2.67(3.47)
TNF-R1, ng/ml	993.81(379.91)	1068.36(501.52)	0.549	1044.27(464.03)
TNF-R2, ng/ml	3261.54(1571.67)	3358.41(1483.82)	0.810	3327.12(1501.10)
TNF-α, ng/ml	0.97(0.59)	1.08(0.74)	0.497	1.04(0.69)
IL-6, ng/ml	2.50(1.39)	3.05(3.15)	0.412	2.87(2.70)
PD-1, ng/ml	229.77(128.53)	295.75(209.82)	0.190	274.42(188.97)
PD-L1, ng/ml	53.03(14.66)	52.06(13.56)	0.793	52.37(13.81)
NfL, pg/ml	16.00(13.56)	12.18(4.43)	0.161	13.83(9.58)
DDD: Second-generation antipsychotics	0.92 (0.67)	0.66(0.68)	0.100	0.75(0.69)
DDD: Mood stabilizers	0.59 (0.37)	0.45(0.28)	0.077	0.50 (0.32)
DDD: Antidepressants	0	0.02(0.17)	0.475	0.01 (0.13)
DDD: Benzodiazepine	0.47 (0.61)	0.63(0.74)	0.325	0.58 (0.69)

DM : diabetes mellitus; HTN : hypertension; CAD : coronary artery disease; HBV/HCV : hepatitis B/C virus; IQ : intelligence quotient; BMI : body mass index; HAM-D : Hamilton depression scale; YMRS : Young mania rating scale; CRP : C-reactive protein; PD-1 : programmed cell death protein 1; PD-L1 : programmed cell death ligand 1; TNF-R1 : tumor necrosis factor receptor type 1; TNF-R2 : tumor necrosis factor receptor type 2; TNF-α : tumor necrosis factor alpha; IL-6 : interleukin-6; NfL : neurofilament light chain; DDD : defined daily dose

*p-value < 0.05

** *p-value* < 0.01

### Correlations between Proinflammatory cytokines/pd system with NfL and clinical features

Correlations between proinflammatory cytokines, PD-1/PD-L1 and clinical features are presented in Table 2. Before FDR correction, positive correlations were observed between TNF-α, TNF-R1, and TNF-R2 levels and the number of episodes requiring admission (TNF-α *r* = 0.79, *p* = 0.023; TNF-R1 *r* = 0.322, *p* = 0.015; and TNF-R2 *r* = 0.310, *p* = 0.020, respectively). Additionally, CRP levels were positively correlated with the total DDD of psychoactive medications used (*r* = 0.308, *p* = 0.013). Furthermore, NfL levels showed positive correlations with PD-1, TNF-R1, and TNF-R2 (*r* = 0.391, *p* = 0.014; *r* = 0.355, *p* = 0.027; and *r* = 0.453, *p* = 0.004). After FDR correction, the only statistically significant association that remained was the positive correlation between NfL and TNF-R2 (FDR-corrected p-value, q = 0.028), which is highlighted in bold in Table 2.

**Table 2:** Correlation between clinical characteristics and pro-inflammatory and PD system markers among OABD patients

Variable	CRP *r* (*p*)	TNF-R1 *r* (*p*)	TNF-R2 *r* (*p*)	TNF-α *r* (*p*)	IL-6 *r* (*p*)	PD-1 *r* (*p*)	PD-L1 *r* (*p*)
Age	-0.047 (0.710)	0.081 (0.522)	0.240 (0.054)	0.030 (0.795)	-0.037 (0.750)	0.228 (0.068)	0.082 (0.514)
Duration of illness	0.030 (0.824)	0.106 (0.436)	-0.007 (0.961)	0.137 (0.272)	-0.121 (0.333)	0.014 (0.920)	0.041 (0.766)
Number of episodes requiring admission	0.028 (0.837)	0.322, (0.015) *	0.310 (0.020) *	0.279 (0.023) *	0.217 (0.080)	0.072 (0.596)	0.219 (0.106)
HAM-D	0.022 (0.863)	-0.014 (0.911)	0.079 (0.534)	-0.030 (0.794)	-0.028 (0.812)	0.033, (0.797)	-0.018 (0.884)
YMRS	0.060 (0.634)	0.050 (0.695)	0.107 (0.395)	-0.151 (0.193)	0.071 (0.541)	0.138 (0.274)	0.030 (0.815)
Psychoactive DDDs	0.308 (0.013) *	0.182 (0.147)	0.114 (0.366)	0.112 (0.337)	0.084 (0.497)	-0.046 (0.718)	0.200 (0.110)
NfL	0.152 (0.356)	0.355 (0.027) *	0.453 (0.004) **	0.195 (0.174)	0.064 (0.657)	0.391 (0.014) *	0.121 (0.463)

CRP: C-reactive protein; PD-1: programmed cell death protein 1; PD-L1: programmed cell death ligand 1; TNF-R1: tumor necrosis factor receptor type 1; TNF-R2: tumor necrosis factor receptor type 2; TNF-α: tumor necrosis factor alpha; IL-6: interleukin-6; HAM-D: Hamilton depression scale; YMRS: Young mania rating scale; DDD: defined daily dose; NfL: neurofilament light chain

*p-value < 0.05

** *p-value* < 0.01

Bold indicates statistical significance after False Discovery Rate (FDR) correction

### Correlations of clinical variables with cognitive outcomes

Correlations between clinical variables and cognitive domains in OABD are presented in Table 3. Significant findings after FDR correction are indicated in bold. A negative correlation was found between age and processing speed. The number of episodes requiring admission was negatively correlated with the composite cognitive score, verbal memory, motor speed, working memory, and processing speed. Years of education were positively correlated with all cognitive domains except motor speed. Body mass index (BMI) was negatively correlated with the composite score, verbal memory, working memory, and processing speed. NfL was negatively correlated with verbal fluency, but this did not remain significant after FDR correction. FRS score was negatively correlated with the composite score, working memory, and processing speed. These significant clinical variables related to cognitive outcomes in OABD underscore the importance of adjusting for these confounding factors when assessing the association between proinflammatory cytokines/PD system and cognitive function.

**Table 3:** Correlations of demographic and clinical variables with cognitive domains in older patients with BD

	Composite score	Verbal memory	Motor speed	Working memory	Verbal fluency	Processing speed	Executive function
	r (p)	r (p)	r (p)	r (p)	r (p)	r (p)	r (p)
Age	-0.163 (0.131)	-0.236 (0.028) *	-0.078 (0.471)	-0.163 (0.131)	-0.130 (0.230)	**-0.290** (0.006) **	0.071 (0.515)
Duration of illness	-0.043 (0.722)	0.061 (0.616)	-0.182 (0.131)	-0.088 (0.467)	-0.040 (0.744)	0.005 (0.970)	-0.142 (0.241)
Number of episodes requiring admission	-0.477 (< 0.001) **	-0.393 (0.001) **	-0.453 (< 0.001) **	**-0.345** (0.003) **	-0.275 (0.021) *	**-0.393** (0.001) **	-0.214 (0.075)
Educational year	0.493 (< 0.001) **	0.409 (< 0.001) **	0.183 (0.128)	**0.362** (0.002) **	0.389 (0.001) **	**0.464** (< 0.001) **	**0.420** (< 0.001) **
BMI	-0.493 (< 0.001) **	-0.493 (< 0.001) **	-0.283 (0.024) *	-0.480 (< 0.001) **	-0.201 (0.113)	-0.426 (< 0.001) **	-0.269 (0.033) *
HAM-D	-0.198 (0.066)	-0.182 (0.147)	-0.212 (0.048) *	-0.073 (0.505)	-0.129 (0.234)	-0.210 (0.051)	-0.126 (0.244)
YMRS	0.104 (0.337)	0.139 (0.198)	0.070 (0.522)	0.188 (0.081)	0.205 (0.057)	0.031 (0.775)	0.108 (0.320)
NfL	-0.266 (0.059)	-0.242 (0.087)	-0.159 (0.265)	-0.174 (0.223)	-0.283 (0.044) *	-0.148 (0.300)	-0.257 (0.069)
FRS score	-0.256 (0.014) *	-0.212 (0.043) *	-0.110 (0.297)	**-0.275** (0.008) **	-0.230 (0.027) *	**-0.297** (0.004) **	-0.087 (0.412)

BMI: body mass index; HAM-D: Hamilton depression scale; YMRS: Young mania rating scale; NfL: neurofilament light chain; FRS: Framingham risk score

*p-value < 0.05

** *p-value* < 0.01

Bold indicates statistical significance after False Discovery Rate (FDR) correction

### Effect of Proinflammatory cytokines/pd system levels on cognitive domains

The variables “number of episodes requiring admission” and “total number of episodes” showed high collinearity; therefore, we used “number of episodes requiring admission” as a proxy as which may help reduce recall bias. The variable “duration of illness” did not show significant associations in either Table 2 or Table 3. Accordingly, in the final regression analysis, adjustments were made for age, sex, and the significant clinical factors identified above—including years of education, BMI, total psychoactive DDD, number of episodes requiring admission, NfL levels, and FRS score—to assess the independent effects of pro-inflammatory cytokines and PD system markers on individual cognitive domains (Table 4).

**Table 4:** Regression model for pro-inflammatory markers and PD system to cognitive function in older patients with BD

	Composite		Verbal memory		Motor speed		Working memory		Verbal fluency		Processing Speed		Executive function	
	B (SE)	*p*	B (SE)	*p*	B (SE)	*p*	B (SE)	*p*	B (SE)	*p*	B (SE)	*p*	B (SE)	*p*
CRP, mg/ml	-0.036(0.043)	0.394	-0.028(0.035)	0.428	0.006(0.036)	0.857	-0.059(0.284)	0.284	-0.028(0.025)	0.247	0.002(0.041)	0.959	-0.030(0.052)	0.560
IL-6, ng/ml	0.073(0.109)	0.501	0.063(0.090)	0.479	0.099(0.091)	0.280	0.146(0.142)	0.303	0.080(0.063)	0.203	-0.072(0.105)	0.495	-0.006(0.133)	0.964
PD-1, ng/ml	-0.002(0.001)	0.033*	0.000(0.001)	0.667	-0.002(0.001)	0.033*	-0.004(0.001)	0.001**	-0.001(0.001)	0.049*	-0.001(0.001)	0.515	< 0.001 (0.001)	0.852
PD-L1, ng/ml	0.008(0.169)	0.618	-0.002(0.014)	0.863	-0.016(0.014)	0.260	0.002(0.022)	0.945	-0.002(0.010)	0.836	-0.018(0.016)	0.266	0.055(0.021)	0.007**
TNF-R1, ng/ml	-0.002(0.001)	0.072	< 0.001 (0.001)	0.623	-0.002(0.001)	0.006**	-0.001(0.001)	0.427	-0.002(0.006)	0.004**	-0.001(0.001)	0.263	-0.001(0.001)	0.586
TNF-R2, ng/ml	0.001(0.000)	0.180	< 0.001 (0.000)	0.916	0.001(< 0.001)	< 0.001**	0.001(0.001)	0.087	0.001(< 0.001)	0.006**	0.001(0.000)	0.094	-0.001(0.001)	0.045*
TNF-α, ng/ml	-0.046(0.348)	0.895	0.099(0.286)	0.728	-0.543(0.292)	0.063	0.046(0.452)	0.918	-0.108(0.200)	0.591	-0.111(0.335)	0.741	0.262(0.424)	0.536
NfL, pg/ml	-0.001(0.020)	0.941	0.008(0.016)	0.634	-0.015(0.017)	0.356	-0.029(0.026)	0.262	-0.013(0.011)	0.252	-0.015(0.019)	0.425	0.036(0.024)	0.130

Model adjusted: age, gender, education year, body mass index, total psychoactive medication defined daily dose, number of episodes requiring admission, C-reactive protein (CRP), interleukin-6 (IL-6), programmed cell death protein 1 (PD-1), programmed cell death ligand 1 (PD-L1), tumor necrosis factor receptor type 1 (TNF-R1), tumor necrosis factor receptor type 2 (TNF-R2), tumor necrosis factor alpha (TNF-α), neurofilament light chain (NfL), Framingham risk score

*p-value < 0.05

** *p-value* < 0.01

Our results revealed a negative association between TNF-R1 levels and motor speed (*B* = − 0.002, *p* = 0.006) and verbal fluency (*B* = − 0.002, *p* = 0.004), whereas TNF-R2 levels showed positive associations with the same cognitive domains (motor speed: *B* = 0.001, *p* < 0.0001, and verbal fluency: *B* = 0.001, *p* = 0.006). Elevated levels of PD-1 were significantly associated with lower scores in composite score (*B* = − 0.002, *p* = 0.033), motor speed (*B* = − 0.002, *p* = 0.033), working memory (*B* = − 0.004, *p* = 0.001), and verbal fluency (*B* = − 0.001, *p* = 0.049). Regarding executive function, PD-L1 showed a positive association (*B* = 0.055, *p* = 0.007), whereas TNF-R2 demonstrated a negative association (*B* = − 0.001, *p* = 0.045). It is worth noting that the associations of PD-1 with verbal fluency (*p* = 0.049) and TNF-R2 with executive function (*p* = 0.045) were near the threshold of statistical significance.

Within the same regression model, a consistent and significant positive association was observed between years of education with all cognitive domains, except for motor speed. The number of episodes requiring admission was negatively associated with verbal memory, motor speed, and working memory. BMI was negatively associated with verbal memory, motor speed, working memory and processing speed. Psychoactive DDD showed no significant association with any cognitive domains after adjusting for other clinical factors. FRS score was specifically negatively associated with verbal fluency (Details shown in Supplemental Table 2).

## Discussion

To our knowledge, this study is the first to explore pro-inflammatory cytokine levels and the PD system in OABD and to assess their relationship with neuroaxonal integrity and cognitive domains. Our results suggest that both PD system markers and pro-inflammatory cytokines are jointly involved in cognitive outcomes in OABD. We found that PD-1 was negatively associated with composite score, especially in motor speed and working memory, while PD-L1 was positively associated with executive function. In addition, TNFR1 exhibited a negative association in motor speed and verbal fluency, while TNFR2 showed a positive association with these two cognitive domains. The findings regarding PD-1 and verbal fluency (*p* = 0.049), and TNF-R2 and executive function (*p* = 0.045), were near the threshold of statistical significance and should therefore be interpreted with caution, as they may reflect weak evidence that warrants further investigation in larger and more homogeneous samples.

The direction of associations of TNFR1 and TNFR2 on motor speed and verbal fluency domains were consistent with the immune characteristics of these two receptors. TNFR2, which is expressed on regulatory T-cells, oligodendrocytes, and astrocytes, plays a role in immunoregulation, neuronal survival, and remyelination. In contrast, TNFR1 is ubiquitously expressed on nearly all cells and promotes cenral nervous system (CNS) inflammation and neuronal demyelination [53–55]. Irradiation studies on mice also revealed that TNFR1−/− and TNF-α−/− deficient animals exhibit enhanced baseline neurogenesis in the hippocampus, whereas the absence of TNFR2 leads to decreased baseline neurogenesis [56]. Studies also suggested that in some autoimmune diseases, such as multiple sclerosis, the expression of TNFR2 on regulatory T-cells might be downregulated and accompanied by an increased level of TNFR1 [57, 58]. In addition, we identified a positive correlation between NfL and TNF-R2 (FDR q = 0.028), suggesting that elevated NfL levels may be linked to remyelination processes during the course of BD, which finding is consistent with previous literature [59, 60]. From this perspective, in our OABD sample, higher levels of TNFR1 were associated with poorer performance in motor speed and verbal fluency, suggesting a potentially detrimental effect of elevated cytokines linked to TNFR1. In contrast, TNFR2 appears to play a role in shaping the neuroinflammatory environment by reducing pro-inflammatory cytokine levels [61]. Accordingly, TNFR2 was positively associated with performance in these cognitive domains, possibly reflecting a compensatory response to neuroinflammatory processes. This interpretation is supported by previous findings that suggest a neuroprotective role of TNFR2 in cognitive function.

To date, no prior research has directly investigated the relationship between PD-1 or PD-L1 and BD. In this study, we explored the relationship between their concentrations and cognitive function in OABD. The interaction between PD-1 and PD-L1 was shown to play a crucial role in suppressing T-cell responses in vivo [35]. PD-1 expression is typically induced on activated T-cells, B-cells, and myeloid cells in response to immune stimulation. Its expression is tightly regulated and increases during chronic antigen exposure, such as is seen in chronic infections or cancer. High levels of PD-1 expression are often associated with T-cell exhaustion, a state in which T-cells lose the capability of function. Nevertheless, the interaction between PD-1 and its ligands, particularly PD-L1, plays a critical role in maintaining immune homeostasis by preventing excessive immune responses [62]. For example, studies involving PD-L1−/− T-cells suggest that PD-L1 expressed on T-cells can downregulate cytokine production, thereby modulating immune activity [33, 63]. Based on these findings, we suggest that elevated PD-1 levels may reflect an ongoing inflammatory response, in conjunction with increased pro-inflammatory cytokines, and were associated with poorer cognitive performance—especially in composite scores, motor speed, and working memory. Interestingly, in the domain of executive function, higher PD-L1 levels may exert a compensatory effect in the context of heightened inflammation, thereby showing a positive association with cognitive performance.

The regulation of PD-1 is influenced by cell-specific factors in addition to the cytokine microenvironment thus resulting in various expression patterns [64]. Although the precise mechanisms remain unclear, there may be crosstalk between the PD system and the TNF signaling pathway. For example, anti-TNF antibodies have been shown to enhance the efficacy of anti-PD-1 treatment in mouse models of melanoma [65]. In addition, the use of clinically available TNF inhibitors in combination with CTLA-4 and PD-1 immunotherapy in mice has been found to alleviate colitis and improve anti-tumor efficacy [66]. In the context of advanced pancreatic adenocarcinoma, the in vitro mechanism of TNFR2 regulation of PD- L1 was investigated, and results suggested that TNFR2 may regulate the expression of PD-L1 via the p65 nuclear factor (NF)-κB pathway [67]. However, these studies have primarily focused on tumor cell models and basic experimental settings. The detailed interactions between the PD system and pro-inflammatory cytokines in BD remain largely unexplored and require further investigation.

A major strength of this study lies in its comprehensive assessment of cognitive aging–related variables within a well-defined OABD population. Another key strength is the inclusion of a wide range of clinically relevant covariates—such as education level, BMI, psychoactive medication load, NfL levels, and number of episodes that with admission—which strengthens the regression models and reduces the risk of confounding. In addition, the diagnosis of BD was made by board-certified psychiatrists using standardized procedures in a tertiary psychiatric hospital, ensuring the validity and reliability of the clinical assessments. However, several limitations should be considered when interpreting the results. First, the sample was recruited from a tertiary psychiatric hospital, likely leading to an overrepresentation of individuals with more severe illness and cognitive impairment, thus limiting the generalizability of the findings to the broader BD population. Second, the study lacked a control group and did not include younger BD patients for comparison, which restricts the contextual interpretation of proinflammatory cytokine and PD-1/PD-L1 levels. Third, the peripheral measurements of proinflammatory cytokines and PD-1/PD-L1 may not accurately reflect central nervous system CNS concentrations, which are more directly relevant to cognitive function. Fourth, although cardiovascular burden was adjusted for using the FRS score, it serves only as a proxy and may not fully capture somatic comorbidities that could influence cognition. The use of a more comprehensive index, such as the Charlson Comorbidity Index, could improve the assessment of frailty and physical health. Fifth, several clinically relevant variables that may affect cognition were unavailable in our dataset. These include early-life learning disorders and prior electroconvulsive therapy—both known to influence cognitive function. Sixth, anticholinergic burden, a more accurate predictor of medication-related cognitive impairment in older adults [68], was not assessed. While total psychoactive DDD was used as a proxy for medication load, it does not account for anticholinergic effects. Furthermore, the lack of data on anti-inflammatory treatments (e.g., NSAIDs, corticosteroids) may have introduced unmeasured confounding, given their potential impact on inflammatory markers. Finally, the cross-sectional design of this study precludes causal inference. Longitudinal studies are needed to investigate the temporal dynamics of inflammation and cognitive decline and to elucidate the underlying mechanisms.

## Conclusion

This study is the first to simultaneously investigate the pro-inflammatory system and the PD-1/PD-L1 pathway in a clinical OABD sample. We found that PD-1 was negatively associated with motor speed and working memory, while PD-L1 was positively associated with executive function. In addition, TNFR1 exhibited a negative association in motor speed and verbal fluency, while TNFR2 showed a positive association with these two cognitive domains. Identifying the specific cognitive domains influenced by these markers may help guide future intervention strategies. Further research is needed to elucidate the mechanisms linking pro-inflammatory cytokines and the PD-1/PD-L1 pathway, particularly in the context of neuroinflammatory processes and the potential neurodegenerative trajectory of BD.

## Supplementary Information

Below is the link to the electronic supplementary material.

Supplementary Material 1

## Data Availability

The data that support the findings of this study are available on request from the corresponding author.
