# Supplementary material for: Effects of proinflammatory cytokines and programmed cell death on cognitive domains in older age patients with bipolar disorder
Source: Ann Gen Psychiatry. 2025 Aug 12;24:48. doi: 10.1186/s12991-025-00591-9 (PMC12341215; doi:10.1186/s12991-025-00591-9)
Supplement: Supplementary file 1 — Supplementary Material 1 [file 12991_2025_591_MOESM1_ESM.docx]

Supplement table1. Cognitive function between sexes in OABD patients

| Z score, Mean (SD) | Male (N=30) | Female (N=57) | P-value |
| --- | --- | --- | --- |
| Composite score | -2.03 (1.18) | -2.64 (1.86) | 0.068 |
| Verbal memory | -1.58 (1.07) | -1.85 (1.25) | 0.318 |
| Motor speed | -0.70 (0.91) | -1.02 (1.17) | 0.195 |
| Working memory | -1.33 (1.56) | -1.37 (1.62) | 0.923 |
| Verbal fluency | -1.85 (0.56) | -2.13 (0.81) | 0.101 |
| Processing speed | -1.66 (0.99) | -2.05 (1.56) | 0.162 |
| Executive function | -0.74 (1.72) | -1.14 (1.74) | 0.304 |

Supplement Table 2. Regression model for clinical and demographic data to cognitive function in OABD ^a^

|  | Composite | | Verbal memory | | Motor speed | | Working memory | | Verbal fluency | | Processing Speed | | Executive function | |
| --- | --- | --- | --- | --- | --- | --- | --- | --- | --- | --- | --- | --- | --- | --- |
|  | **B** (SE) | *p* | B (SE) | *p* | B (SE) | *p* | B (SE) | *p* | B (SE) | *p* | B (SE) | *p* | B (SE) | *p* |
| Age | -0.002(0.040) | 0.952 | 0.009(0.037) | 0.811 | -0.025(0.032) | 0.447 | -0.017(0.057) | 0.771 | 0.007(0.023) | 0.768 | -0.040(0.037) | 0.283 | 0.065(0.042) | 0.116 |
| Sex ^b^ | 0.529(0.337) | 0.116 | 0.257(0.310) | 0.408 | 0.180(0.271) | 0.505 | 1.216(0.477) | 0.011* | -0.069(0.194) | 0.720 | 0.268(0.309) | 0.385 | 0.489(0.348) | 0.160 |
| Educational years | 0.210(0.046) | <0.001** | 0.111(0.043) | 0.009** | 0.013(0.037) | 0.735 | 0.168(0.066) | 0.010* | 0.056(0.027) | 0.037* | 0.146(0.043) | 0.001** | 0.279(0.048) | <0.001** |
| Number of episodes requiring admission | -0.061(0.028) | 0.027* | -0.064(0.023) | 0.005** | -0.085(0.023) | <0.001** | -0.089(0.036) | 0.013* | 0.002(0.016) | 0.896 | -0.048(0.027) | 0.069 | 0.018(0.034) | 0.592 |
| BMI | -0.107(0.031) | 0.001** | -0.063(0.029) | 0.028* | -0.090(0.025) | <0.001** | -0.139(0.044) | 0.002** | -0.017(0.018) | 0.332 | -0.062(0.029) | 0.028* | -0.025(0.032) | 0.432 |
| Psychoactive agents DDD | -0.104(0.202) | 0.607 | -0.086(0.186) | 0.645 | -0.092(0.162) | 0.572 | 0.266(0.286) | 0.353 | -0.014(0.116) | 0.904 | -0.209(0.185) | 0.259 | -0.221(0.209) | 0.289 |
| FRS score | -0.033(0.205) | 0.108 | -0.019(0.169) | 0.268 | -0.002(0.017) | 0.888 | -0.047(0.027) | 0.077 | -0.039(0.012) | 0.001** | -0.008(0.020) | 0.678 | -0.036(0.025) | 0.156 |

BMI: body mass index; DDD: defined daily dose; FRS: Framingham risk score

^a^ Model same as Table 4

^b^ Female as reference

*p-value <0.05

** *p-value* <0.01
